# Literary Notes

**Published:** 1898-12

**Authors:** 


### LITERARY NOTES.

New Brochure.—The Mulford Company's 1898 antitoxin brochure has recently been received. It is an improvement on that of last year, for which there was much demand, and it appears to be a valuable reference book for physicians. The subjects of antitoxin dosage, symptomatic treatment of cases seen late and immunisation are very complete. There is a novel section on official reports on the use of antitoxin which will be highly appreciated. The book is handsomely finished, and is a masterpiece of trade literature. The book will be mailed gratis by the publishers upon receipt of request.

Harold Frederic's Unpublished Story.—Some weeks before his death, Mr. Harold Frederic finished the book on which he had been at work for months past. This last and most notable novel of this brilliant writer—for it is the only manuscript which he left—far eclipses in power any of his earlier stories. Written when his genius had fully matured, he put the best of himself into "The Market Place," as the novel is called, and it is destined to be the book of the year. For marvelous portrayal and analysis of character, for vigor of style, and for the brilliance and faithfulness of its pictures, it is a novel among novels.

The story has to do with the fortunes of a daring speculator, and, incidentally, with the corruption existing among the titled directors of English companies. Though written before the Hooley disclosures, it seems almost a prophecy of this *cause célèbre*. Mr. Frederic's pictures of English society and English country life are the best and most convincing that have yet been given to us by a novelist.

"The Market Place" has been secured by *The Saturday Evening Post*, of Philadelphia, and will appear serially in that weekly, beginning in an early issue.

The Antikamnia Chemical Company, St. Louis, has prepared and will send out to the medical profession in a few days its Calendar for 1899. It is entitled Skeleton Sketches and is constructed on lines similar to previous years, and is by the well-known artist, the late Dr. L. Crusius. Some of the sketches, which we observe in the advance copy kindly sent the Journal, are exceedingly well drawn, among which are "Ein Stein" and "A Rough Rider." These are clever, artistic hits and merit special attention.

The *Dental Practitioner and Advertiser*, published by the Dental Manufacturing Company, of Buffalo, for the past twenty-nine years, was discontinued with its issue for October, 1898. Dr. Theodore G. Lewis was its editor for twenty-two years and Dr. W. C. Barrett has conducted it since the retirement of Dr. Lewis, so it has had but two editors since it was established. It has been an excellent journal, maintaining a high standard for the profession of dentistry, and we shall miss it as an exchange.

---

THE Grosvenor Library, in Buffalo, is establishing a medical department in rooms that are being especially fitted up for that purpose. It is the intention of the managers to equip this branch in the most complete manner and to make it a superb reference medical library, where not only all the latest books and magazines may be found, but the classics in medicine as well.

When the rooms are finished, and it is excepted that they will be ready before the Christmas holidays, they will be thrown open to inspection by the physicians of Buffalo and vicinity, for which purpose a day will be set apart and due notice given.

The location of the Grosvenor Library and the liberality of the management ought to make its medical department a success from the start. Physicians, medical students and others interested may here find an opportunity to consult, read or abstract medical articles between the hours of 10 a. m. and 10 p. m., without disturbance, in an exclusive department especially adapted to the purpose and equipped with writing conveniences.

Contributions to the library of books and magazines will be appreciated by the managers.

---

THE *Canadian Practitioner*, of Toronto, in its issue for November, 1898, makes the announcement that it has agreed to an amalgamation with the *Medical Review*, and that on and after January, 1899, the name of the new journal will be the *Canadian Practitioner and Medical Review*. The editorial staff is not announced, but we tender the management our congratulations and best wishes.

---

THE *International Medical Journal*, of Philadelphia, announces a change in its editorial management. Dr. Walter L. Pyle retires and Dr. Boardman Reed succeeds to the chair. The latter has introduced some new features that are expected to add to the general usefulness of this excellent magazine.

---

THE *Therapeutic Digest* is a new candidate for professional favor that is published at Kansas City, Kan., and is edited by Dr. J. M. Thompson, with Dr. E. H. Todd as assistant. Dr. V. L. Todd is the general manager. The first numbers present a good appearance.

